# AI-Enabled IoT Framework for Leakage Detection and Its Consequence Prediction during External Transportation of LPG

**DOI:** 10.3390/s23146473

**Published:** 2023-07-17

**Authors:** Amiya Dash, Shuvabrata Bandopadhay, Soumya Ranjan Samal, Vladimir Poulkov

**Affiliations:** 1 School of Engineering and Technology, BML Munjal University, Gurugram 122413, India; amiya.dash@bmu.edu.in; 2 School of Physical Sciences, Banasthali Vidyapith University, Sikar 304022, India; shuvabrata@banasthali.in; 3 Faculty of Telecommunications, Technical University of Sofia, 1756 Sofia, Bulgaria; vkp@tu-sofia.bg; 4 Department of Electronics and Communication Engineering, Silicon Institute of Technology, Bhubaneswar 751024, India

**Keywords:** IoT, artificial intelligence, industrial accident, disaster management, computational fluid dynamics (CFD)

## Abstract

An accident during the transport of liquefied petroleum gas (LPG) via a tanker vehicle leads to the leakage of a flammable substance, causing devastation. In such a situation, the appropriate action with the shortest possible delay can minimize subsequent losses. However, the decision-making mechanism remains unable to detect the occurrence of an accident and evaluate its extent within the critical time. This paper proposes an automatic framework for leakage detection and its consequence prediction during the external transportation of LPG using artificial intelligence (AI) and the internet of things (IoT). An AI model is developed to predict the probable consequences of the accident in terms of the diameter of risk contours. An IoT framework is proposed in which the developed AI model is deployed in the edge device to detect any leakage of gas during transportation, to predict its probable consequences, and to report it to the remotely located disaster management team for initiating appropriate action. A prototype of the proposed model is built and its performance is successfully tested. The proposed solution would significantly help to identify efficient disaster management techniques by allowing for quick leakage detection and the prediction of its probable consequences.

## 1. Introduction

Fires involving chemicals and subsequent explosions are very common, as well as the most significant type of industrial accident. Any leakage in the container allows the hazardous and flammable chemicals to mix with external oxygen and, upon reaching the critical mass for ignition, an exothermic oxidation reaction takes place, resulting in fire. Though many precautionary measures are already implemented inside industry premises, the major concern is accidents during the external transport of hazardous or flammable substances [1,2]. The transportation of flammable chemicals is an integral part of industrial activities; flammable substances like petroleum products are largely transported by tankers via roads or railways. As per the statistics from the United Nations, 50% of all industrially transported goods fall under the category of dangerous goods [3]. An accident occurring during the external transport of flammable substances can lead to catastrophic consequences, causing huge losses of life and property [4]. Mechanical faults present in the system, such as damaged couplings and hoses used for loading and unloading LPG on and off tanker trucks, are a major source of fire accidents [5]. The overturning or collision of an LPG-filled tanker vehicle leads to leakage of the flammable substance and subsequent vapor-cloud formation, fire, and, finally, a fireball, causing devastation [6]. The extent of damage caused in the surrounding area, which includes fatalities, injuries with first- and second-degree burns, and severe to moderate damage to property, is directly related to amount of generated heat flux and pressure [7,8]. In such cases, the appropriate action with the least possible delay is the key to minimizing subsequent losses. The leakage of gas or liquid, if not attended to, may lead to fire and explosion within a very short time span. The remedial action may vary from the restoration of the leakage to optimizing resource mobilization to a mass or controlled evacuation, depending on the situation [9]. However, the decision-making mechanism remains unable to evaluate the accident occurrence, the location of the accident site, and its extent within the critical timeframe.

Wireless sensor networks (WSN) and the IoT promise to play a significant role in disaster management; any natural or manmade disaster can be monitored and its consequences can be forecast [10]. Various IoT-based safety protocols are efficiently implemented in the petrochemical industry, as discussed by the authors of reference [11] and the references therein. Du et al., proposed an IoT-based real-time monitoring solution for the presence of toxic gas in an oil depository via a GSM platform [12]. An online fault diagnosis model for the process industry is presented by the authors of reference [13] that includes multiple information collection and knowledge sharing points using IoT. Nivedhitha et al., presented a smart smoke and LPG detection system using the Texas EZ430-RF2500 (http://www.ti.com/lit/ug/slau227e/slau227e.pdf, accessed on 12 September 2012) wireless module [14]. An IoT-supported sensor node with an AT Mega32 and a platinum microwire is used to detect dust particles in a gas leak in its vicinity, as proposed by the authors of reference [15], where ZigBee is used as the last-mile communication link. Majder-Łopatka et al., discussed the effectiveness of electrochemical sensors embedded with mobile devices in fire-control applications [16].

The study in this paper is on the safety of the external transportation of LPG. In the last decade, intelligent transport systems that deploy IoT-based techniques for monitoring road conditions and providing real-time information about an accident have gained tremendous popularity [17,18,19]. The post-accident losses can be minimized by lowering the response time of the support mechanisms, which requires an efficient monitoring system [20]. Significant improvement in the response time has been observed by bringing the data processing closer to the end nodes in IoT clouds using edge- and fog-computing techniques [21,22,23,24]. The consequences of accidents during the transportation of LPG, or any other flammable gas, via tanker trucks may be detected, and their location can be tracked using sensors and IoT technology. 

However, the major concern in such accidents is the vapor or gas of flammable materials that can be carried by the wind for long distances while maintaining a high concentration level. Hence, life and property can be protected from encountering the flammable gas/vapor by determining a safe separation distance, based on which the decision-making mechanism can prepare the evacuation plan [25]. The separation distance must be larger than the risk area, which may be evaluated from the study of flammable gas dispersion characteristics [26]. The external environmental conditions, like air temperature, wind speed, relative humidity, etc., at the accident site are the key factors for evaluating the risk area [27]. Although the experimental method is the classical approach to determining gaseous dispersion characteristics, it is very resource-constrained and cannot be carried out in varied environmental scenarios. Efforts have been made to detect the leakage location and map the risk area using unmanned aerial vehicles (UAVs) and drones [28]. The formation of a sensor network was proposed to detect the toxic boundary around the industrial area [29]. However, those methods may be suitable for static industrial locations, but not for external transport. Computational fluid dynamic (CFD) tools are widely used in industries to evaluate the gaseous dispersion characteristics by approximating a real physical scenario in simulative platforms [30,31,32,33,34,35]. The CFD tools, by reconstructing real-world boundary conditions and efficiently processing complex geometry models, can generate highly accurate results, and hence are accepted in various industry safety processes [36]. However, the accuracy of the CFD model heavily relies on detailed meshes and boundary conditions. As fine accuracy is obtained at the cost of a long simulation time, the CFD tools are considered inefficient in predicting the risk area for the given time-sensitive problem. Artificial intelligence (AI) techniques combined with CFD show a promising potential to improve the efficiency of the prediction model. With fewer condition points calculated with CFD, the patterns between the points may be interpolated using AI [37]. 

Machine learning approaches are found to be the most promising tools for risk assessment and analysis. Yang et al. have proposed a hierarchical Bayesian approach to estimate the risk involved in oil spill accidents [38]. A dynamic Bayesian network model-based approach is discussed by the authors of reference [39] for risk assessment in deep water drilling wells. Vairo et al. proposed a Hidden Markov Model (HMM)-based technique to evaluate the reliability of prediction models and tested it for liquefied natural gas (LNG) bunkers [40].

This paper provides a smart solution for disaster management in hazardous substance transportation using AI and the IoT to detect any accidental leakage of the LPG during transportation and the location of its occurrence, evaluate its probable consequence, and report to the remotely located disaster management team. The major contributions of the paper are listed below.

1. The heat dispersion phenomena during leakage of LPG are studied using theoretical and computational fluid dynamics (CFD) simulation-based approaches. A database is created for the diameter of the risk contour based on the CFD simulation with varied environmental conditions.
2. Based on the database, an AI model is developed to predict the effects of any accidental leakage from an LPG-filled tanker truck in terms of the diameter of the risk contours and the degree of severity (heat flux) for any arbitrary environmental condition.
3. An IoT framework is developed that deploys the proposed AI model at the edge devices in order to convey the accident location and its probable consequences, in terms of the diameter of the risk contour, to the disaster management team immediately.

The proposed solution would help a remotely located disaster management team initiate appropriate action with the least possible delay. The organization of the paper is as follows: the proposed AI model is discussed in Section 2, the proposed IoT framework in Section 3, and the results and discussion in Section 4. Section 5 concludes the paper.

## 2. Proposed AI Model

In this section, an AI model to predict the diameter of the risk contour during leakage of gas from an LPG-filled tanker truck is developed. The following steps have been executed in this process:

### 2.1. Behavioral Study of the Heat Dispersion

As LPG is a highly inflammable gas, any leakage of it has the following probable consequences: (i) jet-fire, which is a fire of turbulent dispersion due to the combustion of flammable substances leaking continuously in a specific direction; and (ii) fireball, which is due to the rapid outflow and ignition of pressurized flammable gases. The behavior of heat dispersion is studied using the “solid-flame” model, which considers the flame as a solid object radiating from its entire visible surface. The heat flux, in terms of Watt/m^2^, produced due to a fire is quantified as [41]:

(1) $$q^{\prime }=SEP_{act}F_{view}\tau_{\alpha }$$

where $SEP_{act}$, $F_{view}$, and $\tau_{\alpha }$, are actual surface emitting power, view factor, and atmospheric transmissivity, respectively. $SEP_{act}$ is a function of the burning rate of the fuel, which is inversely proportional to the difference between the boiling temperature of the fuel and the ambient temperature ($T_{A}$). $F_{view}$ is the fraction of the emitted radiation that reaches the receptor per unit area, which is a function of the distance between the flame and the receptor of the radiation (D) and the velocity of the wind ($u_{W}$). $\tau_{\alpha }$ defines the part of the heat flux absorbed by the air, which is a function of D, and relative humidity of the air ($H_{A}$). The risk contour is the locus of the points receiving the same amount of heat flux. The diameter of the risk contour for a specific value of $q^{\prime }$, $D(q^{\prime })$ is empirically related to the given meteorological data: ambient temperature, velocity of the wind, and relative humidity of the air. The details of the empirical relations are given by the authors of reference [41].

### 2.2. Problem Formulation

The analysis presented in the previous section suggests that the real-time atmospheric condition is the decisive feature in the prediction of the risk contour. Hence, the diameter of the risk contour can be represented as:

(2) $$D\left(q^{\prime }\right)\approx f\left(T_{A},u_{W},H_{A}\right)+\epsilon$$

Here, $f\left(.\right)$ represents an unknown function and ϵ represents the random noise term that captures the contributions of the other parameters that influence the output. The objective of the present work is to infer supervised learning models to approximate the unknown function given in Equation (2) for predicting the numerical value of the risk contour diameter for an arbitrary set of $T_{A},u_{W},H_{A}$. In this work, models are prepared for predicting diameters of the risk contour with heat intensities of 2, 5, and 10 $\mathrm{kW}/m^{2}$ in both jet-fire and fire ball consequences.

### 2.3. Dataset Preparation

**Figure 1:**
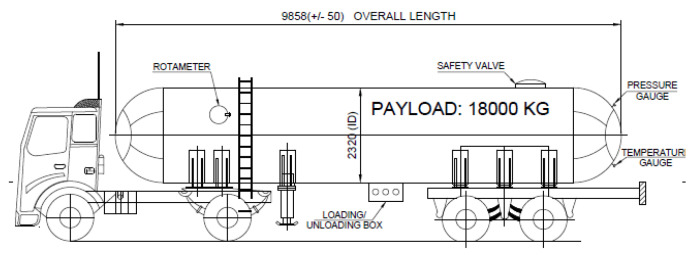
Schematic layout of the tanker truck used in the analysis (all dimensions are in millimeters, drawing not in scale, source: IOCL).

**Figure 2:**
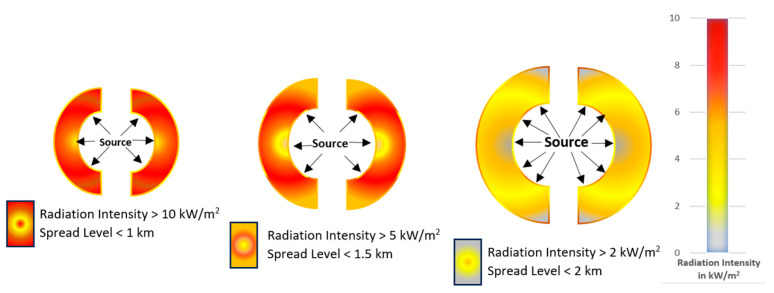
Dispersion analysis diagram for a specific case.

For the dataset, the numerical values of ambient temperature, velocity of wind, and relative humidity of the air were the input attributes, and the corresponding value of risk contour diameter was the response. The dataset was prepared using the CAMEO software suite’s ALOHA (Areal Locations of Hazardous Atmospheres) CFD simulation tool. ALOHA is capable of generating threat zone estimates for various types of hazards for real or potential chemical/hazardous material releases [42]. The ALOHA software tool uses numerical methods and algorithms to predict fluid flow behavior. With a defined geometry of interest, the process divides it into a finite number of discrete regions called “mesh”. For each individual mesh, the fluid flow properties were analyzed by solving the differential equations with respect to initial and boundary conditions with an iterative approach. Using the CFD software tool, a dispersion analysis for the leakage of gas from an LPG-filled tanker truck was carried out with respect to possible explosive accumulations. The dispersion analysis evaluated the probable diameters of risk contours with heat intensities of 2, 5, and 10 kW/m^2^ for a given set of meteorological data triplets ❬$T_{A},u_{W},H_{A}$❭. The schematic layout of the tanker truck used in the analysis is shown in Figure 1. The source data for the dispersion analysis are given in Table 1. Figure 2 depicts the risk diameters with respect to the source for the given examples.

Each simulation constituted a single instance for the subsequent training set. The simulations were carried out with varied possible environmental conditions—ambient temperature, velocity of wind, and relative humidity of the air range between 0 °C and 50 °C, 1 m/s to 12 m/s, and 5% to 99%, respectively—and the outcomes were used as the leveled output. The simulations were carried out for both jet-fire and fireball consequences. All instances were tabulated; the initial three rows of them are shown in Table 2.

### 2.4. Exploratory Data Analysis and Feature Selection

For deeper analytics, the data are described with statistical techniques and represented in Table 3 with their statistical parameters. As the dataset is synthetic data, no null value is present in it.

The CFD process was carried out for a limited number of condition points, and the subsequent training process interpolated the patterns between the points. The interpolation model was deployed to predict the consequences of an accidental leakage on a real-time basis. The best fit hypothesis for said problem was evaluated based on the strength of the relationship between the responses and various input attributes. The strength of the relationship was quantified in terms of correlation measures, which are shown in Table 4. 

For jet-fire, the correlation coefficient was non-zero for all input features and, for fireball, the correlation coefficient was non-zero for the ambient temperature and relative humidity of the air.

### 2.5. Model Selection

Hence, to approximate the unknown function, the hypothesis was taken as a multivariate linear regression function, given by [43]:

(3) $$h_{\beta }\left(m\right)=\beta^{T}m$$

Here, m is the independent input attribute vector given by:

$$m=\left\{\begin{array}{c} {\left[1,T_{A},u_{W},H_{A}\right]}^{T}forjet\text{-}fire\\ {\left[1,T_{A},H_{A}\right]}^{T}for\;fireball \end{array}\right\}$$

And β is the regression parameters vector given by:

$$\beta =\left\{\begin{array}{c} {\left[\beta_{0},\beta_{T_{A}},\beta_{u_{W}},\beta_{H_{A}}\right]}^{T}for\;jet\text{-}fire\\ {\left[\beta_{0},\beta_{T_{A}},\beta_{H_{A}}\right]}^{T}for\;fireball \end{array}\right\}$$

Here, $\beta_{0}$ is the intercept, and $\beta_{T_{A}},\beta_{u_{W}},\beta_{H_{A}}$ are the slope parameters for $T_{A},u_{W},H_{A}$, respectively. The loss function used in the model optimization depends on the square of the difference between the desired value from the dataset and the hypothesis evaluation with the present parameters vector, given by:

(4) $$J\left(\beta |m\right)=\frac{1}{2k}\overset{k}{\underset{i=1}{\sum }}{\left[h_{\beta }\left(m^{\left(i\right)}\right)-D^{\left(i\right)}\right]}^{2}$$

The ith instance of the total k instances present in the dataset is represented with the superscript (i). The optimal solution corresponds to the values of the regression parameters that minimize the loss function:

(5) $$\beta^{c}=arg\;\underset{\beta }{\min}J(\beta |m)$$

The optimum value of the regression parameter vector is obtained using the least-square (LS) process given by:

(6) $$\beta^{c}={\left(M^{T}M\right)}^{-1}M^{T}D.$$

Here, $M={\left\{m^{\left(i\right)}\right\}}_{i=1}^{k}$ is the data matrix and $D={\left\{D^{\left(i\right)}\right\}}_{i=1}^{k}$ is the response vector. For each case of the consequences, the optimum converged regression parameter vectors are found for heat intensity levels 2, 5, and 10 kw/m^2^, respectively.

### 2.6. Model Training and Validation

In the validation process, the total dataset $\Delta =\left\{M:D\right\}$ was split into two disjoint subsets: the training set S and the testing set T, where S∪T=Δ and S∩T=∅. The first part consisted of 80% of the instances used to train the model, and the other 20% were used to validate the model.

The generalization ability of the trained regression models was measured using the coefficient of determination, i.e., $R^{2}$ scores, and the percentage of the error margin. A k-fold (*k* = 5 for this work) cross-validation was implemented to ensure the stability of the trained model. The dataset Δ was split into *k* disjoint subsets with similar size, i.e., $\Delta =\Delta_{1}\cup \Delta_{2}\cup \Delta_{3}\cup \ldots \cup \Delta_{k}$, $\Delta_{i}\cap \Delta_{j}=\emptyset \;\left(i\neq j\right)$. In each trial of cross-validation, the union of (*k*-1) subsets was used as the training set, and the remaining subset was used as the model evaluation testing set. The process was repeated *k* times, with each subset being used as a training set precisely once. The expected results of the 5-fold cross-validation are given in Table 5.

**Figure 3:**
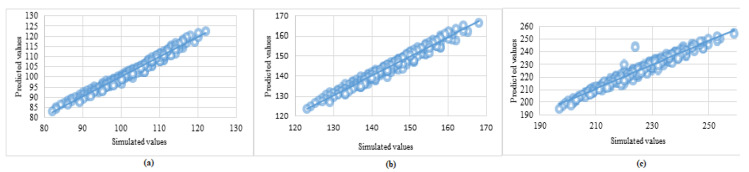
Regression plot, i.e., predicted vs. simulated values (in meters) of diameters of risk contour for jet-fire consequences with heat flux (**a**) $10\mathrm{kW}/m^{2}$ (**b**) $5\;\mathrm{kW}/m^{2}$, and (**c**) $2\;\mathrm{kW}/m^{2}$.

**Figure 4:**
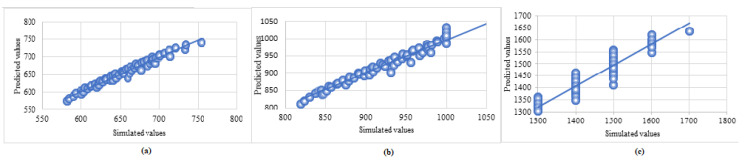
Regression plot, i.e., predicted vs. simulated values (in meters) of diameters of risk contour for fireball consequences with heat flux (**a**) $10\mathrm{kW}/m^{2}$ (**b**) $5\;\mathrm{kW}/m^{2}$, and (**c**) $2\;\mathrm{kW}/m^{2}$.

The final model was created by training with the complete available dataset for predicting the outputs of new data and was saved for operational use in real-time applications. The parameters of the final model are given in Table 6. The regression plot representing the predicted vs. simulated values, along with the respective trend lines for jet-fire and fireball consequences, are shown in Figure 3 and Figure 4, respectively.

## 3. Proposed IoT Framework

In this section, the proposed IoT framework is discussed, which was used to:

- Continuously sense the parts per million (ppm) level of LPG outside the tanker. Based on the measured ppm level, it detected the occurrence of any accidents.
- Upon the occurrence of an accident, predict probable consequences like jet-fire or fireball. For the probable consequence predicted, it evaluated the diameter of risk contours with various degrees of heat flux intensities.
- Report the precise information to the appropriate authority present at the remote location.

**Figure 5:**
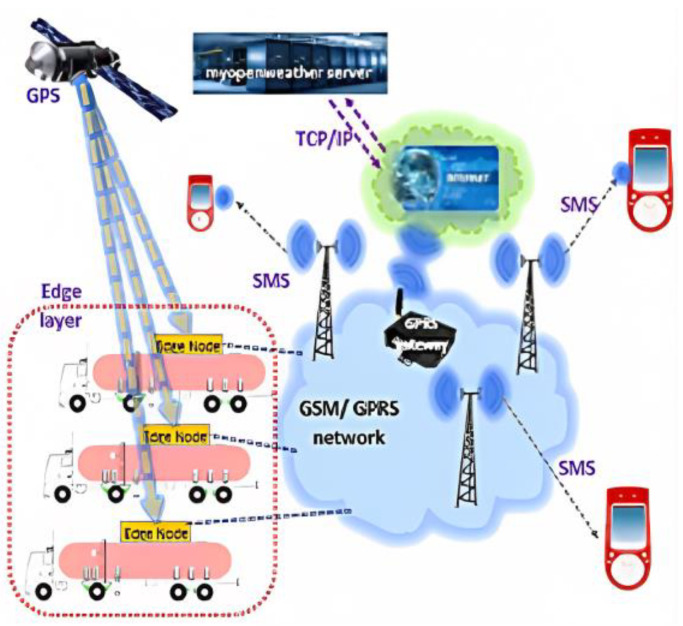
Proposed IoT framework.

The proposed IoT framework, shown in Figure 5, has two functional layers, namely, the edge layer and the network layer. The edge layer comprises edge nodes as its basic building blocks. The edge nodes are the on-board devices installed in each tanker truck availing of the proposed service. The geographically sparsely located edge nodes are connected to the components of the network layer via a wireless link. As the tanker truck moves remotely down the highways, the proposed solution uses the cellular network to establish a communication bridge between the edge layer and the network layer. In this proposed work, the GSM/GPRS network was chosen due to its widest coverage range. The volume of data exchange happening in this application is well within the capacity of the GSM/GPRS network.

As the application is very time-sensitive, the data generated by the sensors needed to be processed on an immediate basis to extract the needed business insight. Hence, the pre-generated AI model was deployed on the edge device to generate essential predictions. With this approach, the system’s time sensitivity improved as it removed the unacceptable latency in the transmission of data to a centralized location.

**Figure 6:**
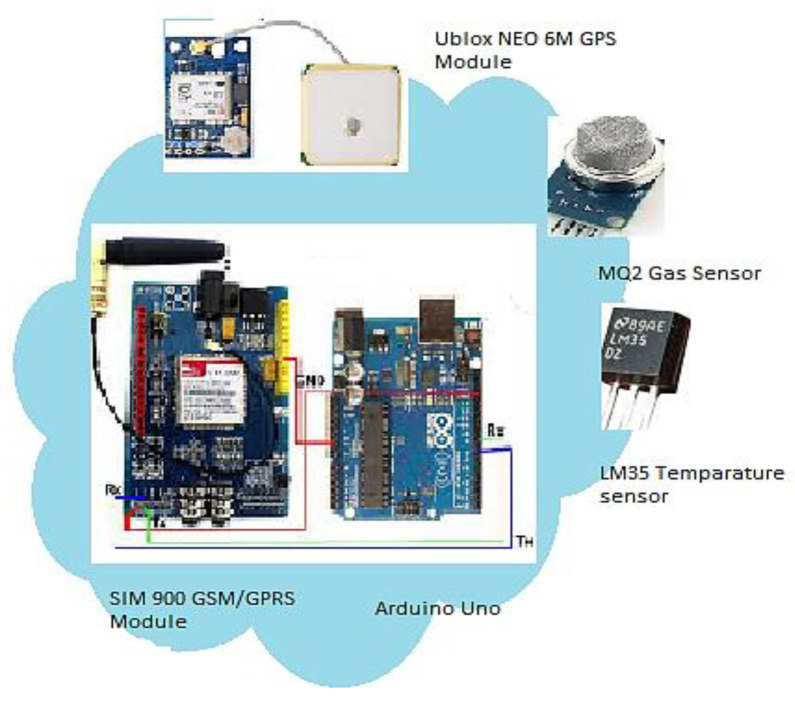
Schematic diagram of edge node.

The hardware of the edge node comprises an Arduino Uno board based on an ATmega3803P microcontroller chip as the central controlling unit. The other modules that have been attached to the central controlling unit are the SIM 900A GSM/GPRS module, the Ublox NEO 6M GPS module, the MQ2 gas sensor, and the LM35 temperature sensor. The schematic of the edge node is shown in Figure 6.

The detection of the occurrence of any accident is performed based on the ppm levels of LPG, smoke, or CO on the outside of the tanker. The leakage of LPG due to any arbitrary cause has the potential to either produce a jet-fire or fireball, resulting in massive destruction. In the proposed edge node hardware, an MQ2 gas sensor is installed at the outlet valve of the tanker to detect any leakage. The sensor output is connected to the analog input pin of the Arduino. The MQ2 sensor is calibrated to report the presence of LPG, smoke, and CO in ambient air in terms of ppm. The system triggers an accident condition when the ppm level of any of the gases exceeds the lower flammable/explosive limit (LFL/LEL) [44]. As the ppm level exceeds the LEL, the presence of an ignition source results in jet fire. With a larger leakage source, the observed ppm value increases towards the upper flammable limit (UFT), resulting in a fireball in the presence of an ignition source. For LPG, the proposed solution triggers the accidental condition with jet fire if the gas concentration level exceeds 2000 ppm, and with fireball if that exceeds 10,500 ppm [45].

After predicting the class of the consequence, the node predicts the diameters of the risk contour using pre-generated linear regression equations. This prediction process needs a real-time metrological data triplet ❬$T_{A},u_{W},H_{A}$❭ to be plugged into Equation (6). The edge node fetches the metrological data triplet by executing the following steps:

The edge node gathers the exact geographical location (latitude and longitude) of the tanker truck via a GPS signaling interface using the Ublox NEO 6M GPS module [46]. The edge node sends an HTTP request to the server of MyOpenWeather through the GPRS network, providing the latitude and longitude information of the accidental site, and waits for the response from the server. On receipt of the server response, the node fetches the real-time metrological data triplet. Using the fetched metrological information, the edge node predicts the risk contour diameter for intensity of heat flux 2, 5, and $10\mathrm{kW}/m^{2}$, respectively, using the regression coefficients of the predicted class of the consequence.

In the reporting process, the end node sends a text message to the authorized mobile numbers via the GSM network. The text message contains the following information: location of the tanker truck that has LPG leakage, ppm level of LPG, probable class of consequence (jet-fire/fireball), and diameters of risk contours. The pseudo-code for the edge node is given in Table 7.

## 4. Prototype Testing and Discussion

**Figure 7:**
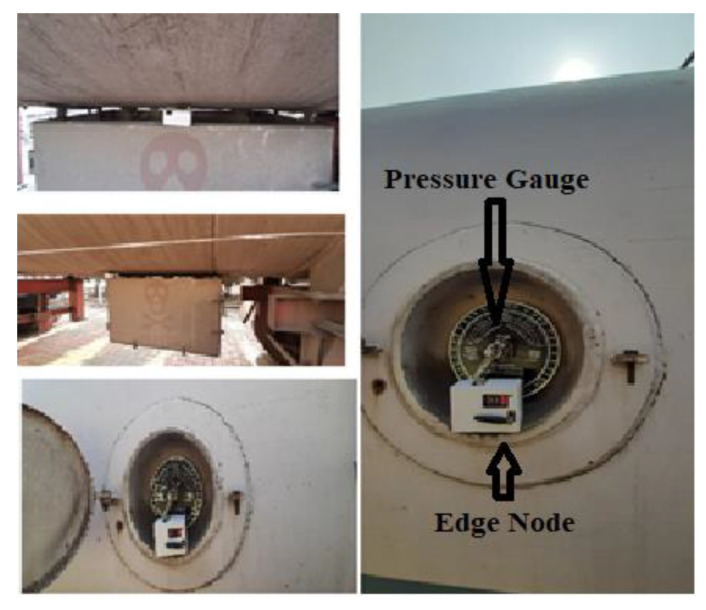
Actual photos of edge node installed in tanker truck for experimental validation.

The prototype of the proposed framework was developed and tested at the LPG bottling Plant, Indian Oil Corporation Limited (IOCL) premises in Malda, West Bengal, India. The possible leakage points of a LPF tanker were the volume/pressure measuring gauge (at the side of the tanker) and the pressure measuring valve mounted at the back of the tanker. The edge node was placed at the volume/pressure measuring gauge. Figure 7 shows the actual pictures of the edge node attached to the tanker truck.

**Figure 8:**
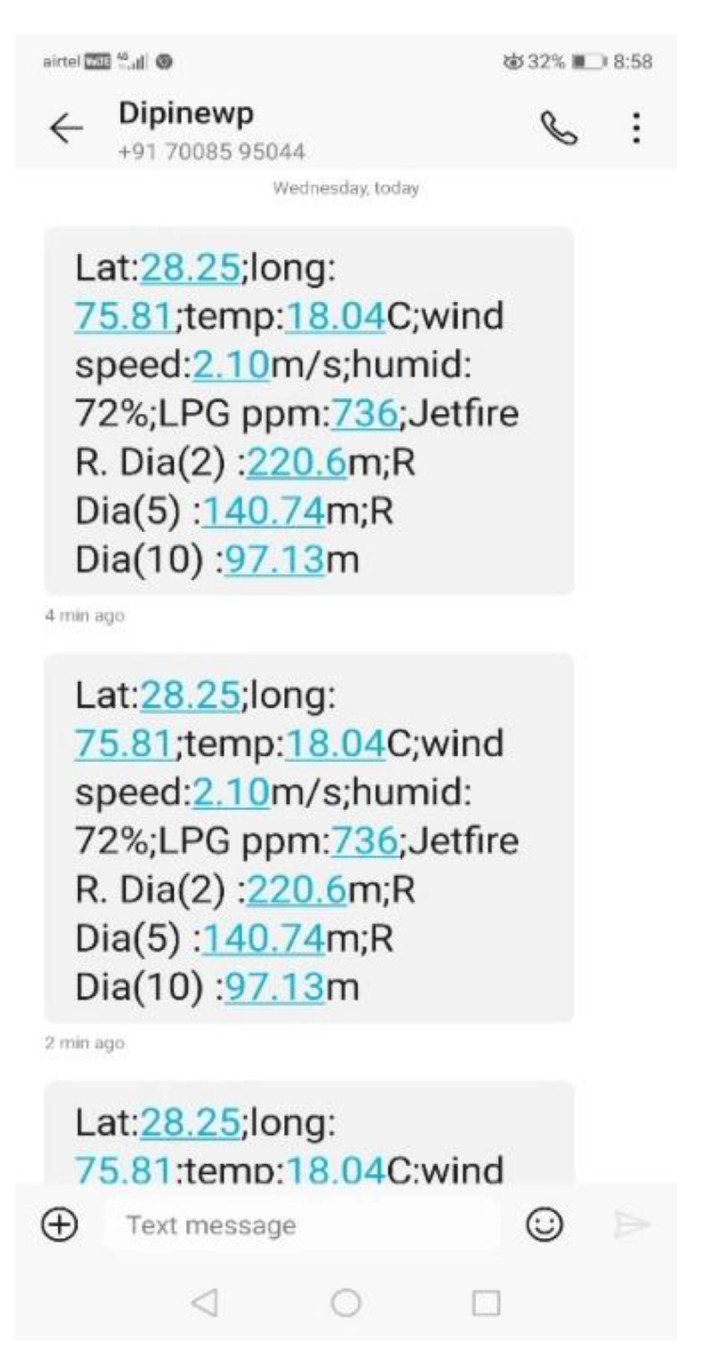
Screenshot of received SMS.

**Figure 9:**
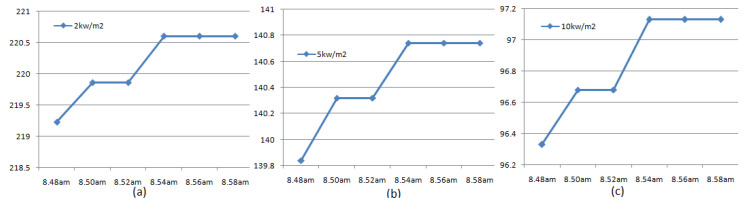
Detected results over time: Time vs. Diameter of risk contour in meters (**a**) $2\;\mathrm{kW}/m^{2}$ (**b**) $5\;\mathrm{kW}/m^{2}$, and (**c**) $10\;\mathrm{kW}/m^{2}$.

The device was powered by a 9-volt battery. To test the performance of the proposed device, LPG gas was intentionally leaked from the gauge/pressure valve following the guidelines of IOCL and instructions from the safety officials at the plant. The attached device started working as instructed in the pseudo-code given in Table 7. The device was triggered and detected leakage when the measured ppm level of LPG exceeded the predefined threshold. Then, it fetched the location information from the GPS, which including the latitude and longitude of the experimental location. The latitude and longitude values were sent to the MyOpenWeather server to fetch the required real-time weather information for the location, i.e., ambient temperature, wind speed, and humidity. Finally, using the predefined model represented in Equation (6), the risk contour diameters were evaluated. With the help of GSM AT commands, the information generated in this process was sent to latitude and longitude in the form of SMS. The information includes: (i) latitude and longitude of the location; (ii) ambient temperature; (iii) wind speed; (iv) humidity; (v) ppm level of LPG; (vi) expected consequence (jet-fire or fire ball); and (vi) diameter of risk contours with heat intensities of 2, 5, and 10 kw/m^2^. The SMS was set to generate periodically with an interval of 120 s. The screen shots of the registered mobile containing a few samples of received SMSs are shown in Figure 8. The test was conducted for nearly 15 min. Figure 9 shows the detected results over time. The predicted values in the SMS were validated with the simulated values and were found to be in close agreement. As this test involves high fire and life risks, the device was not tested with fire.

## 5. Conclusions

This paper proposes an AI-enabled IoT solution for disaster management in the transportation of hazardous substances. The proposed IoT framework immediately reports any leakage of LPG during transportation and its probable consequences to the remotely located disaster management team. The proposed AI model predicts the consequences at the edge device of the IoT network on a real-time basis, eliminating the need for cloud-based CFD analysis. Hence, the proposed solution was found to be suitable for the given time-sensitive application. The prototype of the proposed model was developed and its performance successfully tested at the IOCL LPG bottling plant in Malda, West Bengal, India. The quick detection of the accidental leakage of hazardous substances during external transport and the prediction of probable consequences would greatly assist in finding effective disaster management strategies and save many lives and properties. 

The practical concern of the present study is the reliability and coverage of the cellular network. The performance may improve with the ultra-reliable low latency communication (uRLLC) characteristics of upcoming 5G networks. The fire-proof end product for a given application is another practical concern; the design of a fire-proof outer cover for edge devices is essential before their on-field deployment. In future work, this proposed methodology can be extended to other frequently transported hazardous chemicals. 

## Figures and Tables

**Table 1:** Parameters for dispersion analysis: chemical parameters. Parameters for dispersion analysis: tank parameters.

**Parameters**	**Specifications**
Boiling Point (°C)	>−40
Vapor Density (AIR = 1)	1.5
Specific Gravity (H_2_O = 1):	0.51 to 0.58 at 50 °C
Flammability	Yes
Low Explosive Level (LEL)	1.8%
Upper Explosive Level (LEL)	12.8%
Acute Exposure Guideline Level	
AEGL-1 (10 min)	10,000 ppm
AEGL-2 (10 min)	17,000 ppm
AEGL-3 (10 min)	33,000 ppm
Immediately dangerous to life or health (IDLH) air concentration	19,000 ppm
**Parameters**	**Specifications**
Tank Diameter	2.320 m
Tank Length	9.858 m
Tank Contained	Liquid
Internal Storage Temperature	$30{\;}^{{}^{\circ}}C$
Chemical Mass in Tank	18 tons
% of Tank full	88%

**Table 2:** Diameter of risk contour (in meters) for different set of data input ❬$T_{A},u_{W},H_{A}$❭.

Input Parameters			$\mathrm{Output}\;\mathrm{Parameter}\;(\mathrm{Diameter}\;\mathrm{of}\;\mathrm{Risk}\;\mathrm{Contour}\;(m)\;\mathrm{with}\;\mathrm{Heat}\;\mathrm{Intensity}\;(\mathrm{kW}/m^{2}))$					
			Jet-Fire			Fireball		
*T_A_* (°C)	$u_{W}\;\;\left(m/s\right)$	$H_{A}\;(\%)$	2	5	10	2	5	10
30	4	50	98	141	219	643	908	1400
40	4	70	93	135	210	612	864	1300
50	8	20	102	144	219	637	898	1400

**Table 3:** Description of dataset with statistical parameters.

Parameters	*T_A_* (°C)	$u_{W}\;\left(m/s\right)$	$H_{A}$ (%)	Diameter of Risk Contour (m)					
				Jet-Fire (kW/m^2^)			Fireball (kW/m^2^)		
				10	5	2	10	5	2
Mean	25	6.25	50	102.6	144.9	224.3	675.9	951.1	1497
S.D.	17.11	4.15	31.57	8.36	8.83	12.7	71	95.86	184.5
25th percentiles	10	3.25	20	97	139	215	627	885	1400
50th percentiles	25	6	50	103	145	224	662	935	1500
75th percentiles	40	9	80	109	151	233	696	982	1700

**Table 4:** Correlation measure table.

Parameters	Jet-Fire (kW/m^2^)			Fireball (kW/m^2^)		
	10	5	2	10	5	2
***T_A_* (°C)**	−0.61	−0.73	−0.78	−0.44	−0.46	−0.38
$u_{W}\;\left(m/s\right)$	0.69	0.49	0.33	~0	~0	~0
$H_{A}$ **(%)**	−0.35	−0.45	−0.49	−0.67	−0.66	−0.65

**Table 5:** Expected values of 5-fold cross-validation evaluation results.

Evaluation Parameter	$\mathrm{Jet}\text{-}\mathrm{Fire}(\mathrm{kW}/m^{2})$			$\mathrm{Fireball}\;(\mathrm{kW}/m^{2})$		
	10	5	2	10	5	2
$R^{2}$ score	0.975	0.96	0.95	0.97	0.95	0.88
% of error margin	4.37	4.39	4.52	6.32	6.12	14.3

**Table 6:** Final model parameters: Jet-fire, *fireball*.

Parameters	$\mathrm{Jet}\text{-}\mathrm{Fire}(\mathrm{kW}/m^{2})$			$\mathrm{Fireball}\;(\mathrm{kW}/m^{2})$		
	10	5	2	10	5	2
$\beta_{0}^{c}$	106.94	155.39	244.44	748.50	1051.7	1653.2
$\beta_{T_{A}}^{c}$	−0.296	−0.374	−0.578	−2.005	−2.777	−4.514
$\beta_{u_{W}}^{c}$	1.396	1.038	1.002	NA	NA	NA
$\beta_{H_{A}}^{c}$	−0.103	−0.139	−0.216	−0.750	−1.018	−1.706

**Table 7:** Pseudo-code for Edge node.

**Require:** LPG leakage monitoring and alerting with location and probable consequences. **Ensure:** Real-time detection of accident, prediction of probable risk contour using real-time environmental data and sending of alert message with minimal delay. 1: **Define** API key of MyOpenWeather server 2: **Define** Calibration parameters of MQ2 gas sensor 3: **Define** Registered SIM number(s) to which the alert message to be sent 4: **Define** Arduino Uno GPIO pins for MQ2 gas sensor, LM35 temperature sensor, SIM900 GSM/GPRS module, Ublox NEO 6M GPS module. 5: PPM←PPM value of LPG TEMP←Skin temperature of tanker truck 6: Set threshold PPM value of LPG for imitating accident condition: Th 7: Switch on the system and ensure GSM network connectivity, signal strength after inserting a valid SIM. **Infinite Loop:** 8: Read the PPM values of MQ2 gas sensor 9: **If** (PPM > Th) 10: Initiate accident condition. 11: Read the location of the device (Latitude and longitude) using GPS module. 12: Send http GET request to the Open weather map server with location information via GSM/GPRS network and wait for response status. 13: **If** (response status = = OK) 14: Prepare the metrological data vector from received server data $m={\left[1,T_{A},u_{W},H_{A}\right]}^{T}$ 15: Read LM35 temperature sensor reading TEMP 16: **If** TEMP > 65 °C Compute diameter of risk contour using Equation (6) with regression parameters of Fireball model 17: **Else** Compute diameter of risk contour using Equation (6) with regression parameters of Jet-fire model 18: Send the SMS text to the registered number containing the information of accident location, LPG PPM level, Diameter of risk contour 19: **End loop**
